# Evaluation of Therapeutic Options in Cervical Intraepithelial Neoplasia: A Narrative Review and Clinical Perspective

**DOI:** 10.3390/jcm15031162

**Published:** 2026-02-02

**Authors:** Ecaterina Tomaziu-Todosia Anton, Cǎtǎlina Ionescu, Gabriel Dăscălescu, Gabriel-Ioan Anton, Daniela Roxana Matasariu, Cristina Albert, Ioana-Sadiye Scripcariu, Mihaela Tomaziu-Todosia, Alin Ciobîcă, Demetra Gabriela Socolov

**Affiliations:** 1 Grigore T. Popa University of Medicine and Pharmacy, University Street, No. 16, 700115 Iasi, Romania; tomaziu-todosia.ecaterina@d.umfiasi.ro (E.T.-T.A.); anton.gabriel-ioan@d.umfiasi.ro (G.-I.A.); daniela.matasariu@umfiasi.ro (D.R.M.); mihaela.tomaziu@gmail.com (M.T.-T.); demetra.socolov@umfiasi.ro (D.G.S.); 2 Department of Obstetrics and Gynecology, Clinical Hospital of Obstetrics and Gynecology Cuza Voda, Cuza Voda Street, No. 34, 700038 Iasi, Romania; 3 Doctoral School of Biology, Faculty of Biology, “Alexandru Ioan Cuza” University of Iași, 700505 Iasi, Romania; gabidascalescu2001@gmail.com; 4 “Ioan Haulica” Institute, Apollonia University, 700511 Iasi, Romania; albident72@yahoo.com (C.A.); alin.ciobica@uaic.ro (A.C.); 5 Department of Biology, Faculty of Biology, “Alexandru Ioan Cuza” University of Iasi, 700505 Iasi, Romania; 6 “Olga Necrasov” Center, Department of Biomedical Research, Romanian Academy, 700506 Iasi, Romania

**Keywords:** cervical intraepithelial neoplasia (CIN), cervical dysplasia, therapeutic management, LEEP, conization, fertility preservation

## Abstract

**Background:** Cervical intraepithelial neoplasia (CIN) represents a precancerous condition whose effective management is crucial for preventing invasive cervical cancer, a disease that remains a leading cause of cancer-related mortality among women worldwide. The long pre-invasive phase of cervical carcinogenesis and the availability of effective screening and treatment procedures make CIN a largely preventable and curable entity. **Objectives:** This review aimed to analyze therapeutic options applied in CIN, correlating interventions with lesion grade and guideline recommendations, in order to outline a management model adapted to the Romanian clinical setting. **Materials and Methods:** A structured narrative review of 20 published articles addressing cervical intraepithelial neoplasia (CIN 1–3) published between 2021 and 2023 was performed. Relevant studies were identified through a targeted literature search and analyzed descriptively. This study synthesized data from the recent literature and international clinical guidelines to identify management trends and context-specific adaptations. **Results:** Extracted variables included lesion grade, reported therapeutic approach (surveillance, excisional, or ablative treatment), reproductive considerations, and patient compliance, with international guidelines used as reference standards. Across the reviewed studies, excisional procedures (conization and LEEP) were predominantly reported for high-grade neoplasia (CIN 2–3), while low-grade lesions (CIN 1) were managed either conservatively or through close surveillance. Treatment decisions described in the literature were strongly influenced by patient age, fertility preservation needs, and obstetric history. Overall, management approaches reported in Romanian and international studies were broadly aligned with current guideline recommendations, although variations were observed in the expectant management of younger patients. **Conclusions:** The findings emphasize the importance of individualized management in cervical dysplasia, integrating lesion characteristics with patient-specific factors. While international guidelines provide a robust framework, their adaptation to the Romanian healthcare context should prioritize patient education, compliance, and structured post-treatment follow-up strategies.

## 1. Introduction

Cervical cancer remains one of the leading causes of cancer-related mortality among women worldwide, with an estimated 660.000 new cases and 350.000 deaths annually, according to the World Health Organization (WHO) [1]. Globally, it is the third most common cancer overall and the sixth most frequent malignancy affecting women in developed countries. Importantly, cervical neoplasia is a largely preventable condition due to its long pre-invasive phase and the availability of effective screening procedures [2]. The incidence of this disease, as with most medical conditions, is closely tied to the strength of a nation’s healthcare infrastructure and the resources allocated to large-scale screening and treatment programs.

The diagnosis and risk stratification of CIN rely on a well-defined sequence of investigations, including cytology, HPV testing, and colposcopy with biopsy. The primary goal is to accurately grade the lesion (CIN 1, 2, or 3) to guide appropriate management strategies, which can range from surveillance to excision, thereby preventing progression to invasive disease [3]. Screening plays a central role in this context, representing a cornerstone of public health initiatives aimed at detecting precancerous lesions or early-stage cancers [4]. By enabling early intervention, screening significantly reduces both incidence and mortality [5]. The ultimate goal is to identify lesions before they progress to invasive disease, thereby allowing treatment to be administered at a stage where success rates are highest [6].

Globally, widespread implementation of screening has led to a marked decline in both incidence and mortality from cervical cancer. According to WHO statistics, these rates have decreased by more than half since the 1960s [7]. Shaw (2000) [8] highlights the case of Canada, where the introduction of the Papanicolaou (Pap) test over 50 years ago was instrumental in reducing the disease burden. Although Canada’s incidence rates in the early 2000s were lower than those observed in many other Western nations, they remained higher than those of Scandinavian countries and Iceland, where highly organized, population-wide screening programs were implemented [8]. More recently, Philp et al. (2018) [9] reported that both incidence and mortality have continued to decrease in Canada. The authors attribute this success to the dual impact of effective cervical cytology screening and the integration of HPV vaccination into public health policy. Specifically, between 1972 and 2006, the Pap test contributed to a 58% reduction in incidence and an 83% reduction in mortality from cervical cancer in Canada [9].

In Romania, however, the therapeutic approach to cervical dysplasia continues to be inconsistent. Clinical practice is often influenced by the availability and adherence to standardized guidelines, the level of clinical expertise among specialists, and the specific institutional context in which patients are treated. This variability underscores the need for critical evaluation of therapeutic strategies as they are applied in real-world clinical settings. Assessing current practices and comparing them with international recommendations is essential to improving the quality and consistency of medical care provided to women with cervical dysplasia.

Against this background, the present study seeks to evaluate therapeutic options in cervical dysplasia through a retrospective case series, analyzed from a clinical perspective. By examining the decision-making processes of healthcare professionals and correlating these with established international guidelines, the study aims to highlight both effective practices and gaps in current approaches. The emphasis is placed on the personalization of medical care, with the overarching goal of aligning local therapeutic strategies more closely with global standards and, ultimately, improving patient outcomes.

## 2. Materials and Methods

The present analysis was designed as a structured narrative review, focusing on the therapeutic management of CIN. This study did not involve original patient-level data but was based exclusively on the analysis of previously published studies addressing both the natural history and the treatment of cervical dysplasia, with particular emphasis on low-grade (CIN 1) and high-grade lesions (CIN 2 and CIN 3).

A targeted literature search of the scientific literature was performed to identify relevant publications. Major biomedical databases were consulted, including PubMed/MEDLINE, BioMed Central (BMC), the American Journal of Obstetrics and Gynecology (AJOG), and the Journal of the American Medical Association (JAMA). Additional peer-reviewed articles were identified through manual cross-referencing of relevant publications. The search strategy employed a combination of controlled vocabulary (MeSH terms) and free-text keywords, using Boolean operators to enhance search sensitivity. The principal search string was as follows:

(“cervical intraepithelial neoplasia” OR “CIN” OR “cervical dysplasia”) AND (“management” OR “treatment” OR “therapy” OR “surveillance”) AND (“regression” OR “progression” OR “recurrence” OR “persistence”) AND (“HPV” OR “Human papillomavirus”).

Searches were refined by applying filters for publication years 2021–2023, English language, and peer-reviewed journals. The final selection comprised 20 articles that were considered relevant to the objectives of this review.

Eligibility criteria were applied at the study level, requiring that the included publications reported histopathologically confirmed CIN, a clearly described therapeutic approach, and reported clinical outcomes over time. The exclusion criteria included incomplete methodological reporting, unclear diagnostic classification, or insufficient outcome description. The included studies encompassed heterogeneous designs (prospective cohorts, retrospective case series, meta-analyses, and modeling studies), reflecting the diversity of available evidence.

For each study, the following data were extracted and systematically tabulated: year of publication, type and grade of cervical lesion under investigation, therapeutic strategy applied (active surveillance, ablative or excisional treatment, HPV vaccination, and cytological or HPV-based monitoring), rate of spontaneous regression, rate of progression to more severe lesions (e.g., CIN 3 or invasive carcinoma), and post-treatment recurrence. Data synthesis was performed descriptively and supported by graphical visualization, which illustrated both the distribution of studies according to lesion grade and predominant therapeutic approach, as well as the average rates of regression and progression across CIN 1, CIN 2, and CIN 3 lesions.

Although the PRISMA (Preferred Reporting Items for Systematic Reviews and Meta-Analyses) guidelines represent the current gold standard for systematic reviews, this work was intentionally designed as a narrative–descriptive review rather than a formal systematic review. This choice was motivated by the relatively small and recent body of literature specifically addressing therapeutic decision making in CIN from 2021 to 2023, as well as the heterogeneity of study designs (ranging from clinical trials to retrospective analyses and modeling studies). Because the primary aim of this work was not to pool effect sizes or perform meta-analysis, but rather to critically contextualize therapeutic options and evaluate their alignment with international guidelines in light of local practice, the structured descriptive review format was considered the most appropriate and scientifically justified. Nevertheless, the search strategy and inclusion criteria were designed to maintain a high degree of transparency, reproducibility, and methodological rigor.

Each study was analyzed with respect to clinical parameters, including patient age and reproductive status, histopathological grade of lesion, presence of HPV infection (when reported), therapeutic strategy chosen, rationale for therapeutic decision (including fertility preservation when relevant), and treatment outcome with subsequent evolution. The extracted data were then compared with the latest international evidence-based guidelines, notably the ASCCP 2019 recommendations and the ESGO 2020 consensus, in order to assess the degree of compliance with best practice standards. This comparative evaluation allowed the identification of both consistent patterns and deviations from standardized care, highlighting the role of clinical context and personalization of treatment decisions.

To enhance methodological rigor, data extraction and interpretation were independently reviewed by two investigators, with discrepancies resolved by consensus or third-party adjudication when necessary. Romanian legislative and healthcare framework considerations were incorporated descriptively, where relevant to therapeutic decision-making. Graphical summaries were generated for illustrative purposes only, using Microsoft Excel.

This work also incorporated relevant Romanian legislative frameworks, while statistical analysis and graphical representations were performed using Microsoft Excel 2019 (Microsoft Corp., Redmond, WA, USA).

Through this methodological framework, this review provides a comprehensive and nuanced perspective on the therapeutic management of cervical dysplasia, integrating published evidence with clinical considerations that directly impact patient outcomes.

## 3. Results

**Figure 1:**
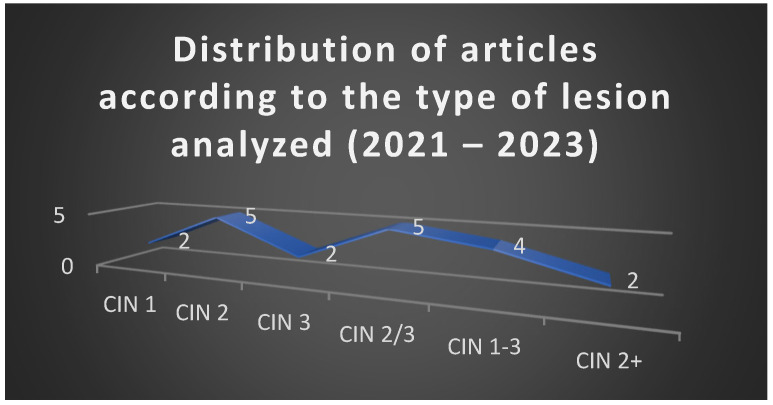
Distribution of articles according to the type of lesion analyzed (2021–2023).

The analysis of the 20 selected articles published between 2021 and 2023 revealed a balanced representation of studies addressing different grades of cervical intraepithelial neoplasia, with a predominance of investigations on CIN 2 and CIN 2/3 lesions. Figure 1 illustrates this distribution, confirming the central position of CIN 2 in current research, reflecting the ongoing debate regarding the optimal balance between conservative and excisional strategies in this intermediate category.

### 3.1. Natural History and Biological Behavior of CIN

The understanding of CIN progression and regression patterns builds on foundational analyses, such as that of Ostör (1993), who systematically reviewed the natural history of these lesions [10].

The reviewed studies consistently reported a favorable natural history for CIN 1. Across the eight articles dedicated to this lesion type, spontaneous regression rates were high (70–72%), while the risk of progression remained minimal (1.5–2%). These findings strongly support the use of active surveillance in compliant young women.

To contextualize our findings within current clinical practice, a comparative overview of regression and progression patterns across CIN grades and their corresponding management recommendations according to the 2019 ASCCP and 2020 ESGO guidelines is presented in Table 1.

In contrast, CIN 2 was the most extensively analyzed, reported in 16 articles, with results highlighting substantial heterogeneity. Spontaneous regression varied widely, from 16% to 77%, depending on patient age, HPV genotype, and follow-up protocols. Progression rates ranged between 9.3% and 36.4%, reflecting the biological variability of these lesions. This reinforces CIN II as the most controversial category, situated between low-grade lesions with high regression potential and high-grade lesions requiring intervention.

CIN 3, the most severe grade, was investigated in 12 specific studies and in an additional six that included combined categories (CIN 2/3 or CIN 1–3). No cases of true spontaneous regression were documented. Instead, the primary clinical concern was recurrence after excisional treatment, particularly the Loop Electrosurgical Excision Procedure (LEEP) or conization, where rates ranged from 4% to 12.5%. Recurrence was strongly associated with positive resection margins and persistence of high-risk HPV infection.

Table 2 summarizes the distribution of lesions and therapeutic strategies across the analyzed articles.

### 3.2. Comparative Regression and Progression Rates

**Figure 2:**
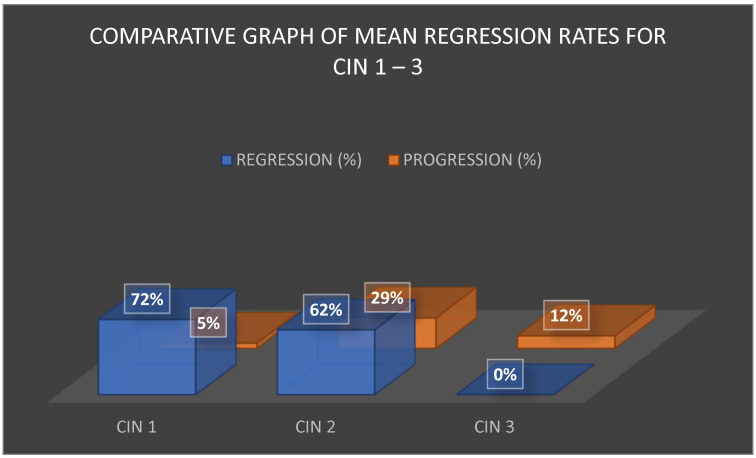
Comparative graph of mean regression rates for CIN 1–3.

The comparative analysis of regression rates across CIN 1, CIN 2, and CIN 3 is shown in Figure 2. CIN 1 demonstrated the most favorable outcomes, with regression exceeding 70% and progression rates averaging around 5%. CIN 2 lesions displayed a moderate regression rate of approximately 63% but also carried a significant progression risk of 25–33%, particularly in women over 30 years or those with persistent HPV infection. CIN 3 was characterized by the absence of reported spontaneous regression, with post-treatment progression or recurrence estimated at 12%, again strongly influenced by HPV persistence and surgical margin status.

These differences in biological behavior support a tailored approach to management. For CIN 1, the predominance of spontaneous regression supports conservative strategies. For CIN 2, clinical decisions require a balance between preventing overtreatment and mitigating progression risk, whereas CIN 3 mandates definitive intervention.

### 3.3. Therapeutic Strategies Reported in the Literature

**Figure 3:**
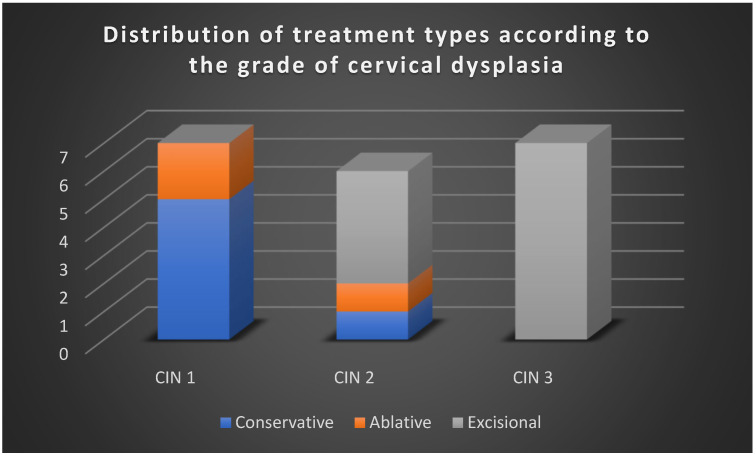
Distribution of treatment types according to the grade of cervical dysplasia (CIN 1–3).

The reviewed studies emphasized four principal management strategies (Figure 3):

- Active surveillance, used predominantly for CIN 1 and, in selected cases, CIN 2, especially in younger women with strong compliance.
- Excisional treatment (LEEP, conization), the mainstay of therapy for CIN 2/3 and CIN 3, particularly when risk factors such as HPV 16/18 infection, positive margins, or advanced age were present.
- Adjuvant HPV vaccination, applied either post-treatment or during surveillance, especially in patients with recurrent CIN 1, suggesting a role in reducing reinfection and recurrence risk.
- Triage and monitoring with HPV testing and cytology, frequently employed to avoid unnecessary procedures, particularly in low-grade lesions.

The clinical evolution data are summarized in Table 3. CIN 1 lesions, monitored under surveillance, exhibited ~ 70% regression within 1–2 years. CIN 2 lesions progressed to CIN 3 in 8–16% of cases, depending on HPV status and vaccination history. CIN 2/3 treated by excision showed a recurrence rate of 5–7% over five years, with a higher risk in HPV-positive cohorts. CIN 3, even after treatment, carried a persistently elevated risk for subsequent anogenital cancers (anal, vaginal, and vulvar), which gradually declined but remained significant over time.

**Figure 4:**
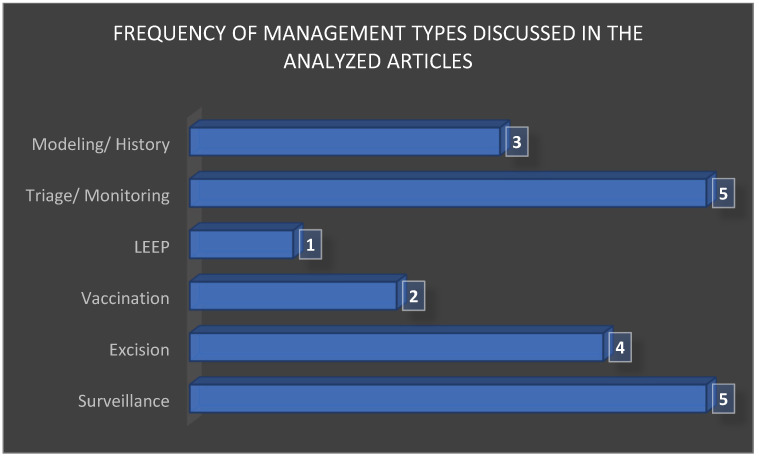
Frequency of management types discussed in the analyzed articles (surveillance, excision, vaccination, and monitoring).

After synthesizing the therapeutic strategies across the 20 included studies, it became evident that management options were not distributed evenly. Active surveillance and excisional procedures (LEEP and conization) were the most frequently reported interventions, reflecting their central role in current clinical decision making. Adjuvant HPV vaccination was less commonly investigated yet consistently highlighted for its potential to reduce recurrence rates, while HPV-based monitoring and cytological triage appeared as complementary strategies to personalize follow-up and avoid overtreatment. The relative frequency of these management approaches is summarized in Figure 4, which illustrates the predominance of excisional procedures and surveillance, followed by molecular and vaccination-based adjuncts.

### 3.4. Clinical Implications

Overall, excisional interventions remain the gold standard for high-grade lesions (CIN 2/3 and CIN 3), yet emerging evidence supports surveillance as a safe alternative in carefully selected CIN 2 cases, particularly in younger women and in the context of HPV vaccination and molecular triage testing (e.g., p16/Ki-67). The use of adjuvant vaccination after treatment has demonstrated benefits in reducing recurrence rates, and its integration into follow-up protocols is increasingly supported by the current literature.

The therapeutic decision-making process was found to align broadly with international guidelines (ASCCP, 2019; ESGO, 2020), with surveillance favored in CIN 1 and excisional treatment predominant in CIN3. For CIN 2, the choice of therapy varied, reflecting the tension between preserving fertility and minimizing oncological risk. Fertility considerations were especially influential: conservative options were prioritized for women of reproductive age, whereas postmenopausal women were more often treated aggressively. Patient compliance emerged as a decisive factor, with successful surveillance dependent on reliable adherence to follow-up, while poor compliance increased progression risk.

Collectively, these findings highlight the complexity of managing cervical dysplasia. The variability in regression, progression, and recurrence rates underscores the importance of individualized care, balancing oncologic safety with fertility preservation and patient-centered decision making.

To complement the aggregated findings presented in Figure 1, Figure 2, Figure 3 and Figure 4, a detailed synthesis of the 20 analyzed studies is provided in Table 4. This table outlines, for each article, the type of lesion studied, the therapeutic strategies applied, additional reported interventions, and regression, progression, or recurrence rates. Such a structured overview enhances transparency and allows the reader to appreciate both the convergences and discrepancies among individual studies, which form the basis of the comparative analysis presented above.

In addition, a summarized overview of clinical outcomes by CIN grade is presented in Table 5, aggregating data across the reviewed studies to highlight regression, progression, and recurrence rates for each lesion grade (CIN 1, CIN 2, CIN 2/3, and CIN 3). This summary table provides a concise comparative perspective, allowing the reader to quickly assess overall trends while maintaining reference to the detailed study-level data in Table 4.

## 4. Discussion

The results of this study demonstrate a strong alignment between the therapeutic approaches and current international guideline recommendations (ASCCP and ESGO), particularly in cases of moderate to severe dysplasia (CIN 2–3), where excisional treatment remains the gold standard. Therapeutic decisions generally aligned with international recommendations; however, local practice shows variability influenced by patient factors and resource availability. This observation is consistent with the existing literature, which shows that the spontaneous regression rate of CIN 1 is high (over 60%), supporting guideline-based active surveillance, though uptake in local practice varies due to clinician and patient factors.

The analysis also underscored the influence of several factors, such as patient age, presence of high-risk HPV, obstetric history, and available healthcare resources, on the choice of therapeutic modality.

These findings reinforce that treatment should not only follow standardized guidelines but also be personalized to the patient’s clinical and socio-economic context.

### 4.1. The Diagnostic Pathway for Precancerous Lesions

While international guidelines provide a clear framework for CIN management, our review highlights variability in adherence within the Romanian context. For example, although ASCCP and ESGO recommend active surveillance for young women with CIN 1 and selected CIN 2 lesions, excisional treatments are sometimes used more frequently in Romanian practice, particularly in older or postmenopausal women. It is paramount to distinguish the diagnostic workflow for CIN from that of invasive cervical cancer. While this study focuses exclusively on the management of precancerous lesions, the definitive diagnosis of CIN is established through a stepwise process that begins with an abnormal screening test (cytology and/or HPV test) [30] and culminates in colposcopic examination and directed biopsy for histopathological confirmation [31,32]. This process determines the grade of dysplasia (CIN 1, 2, or 3) and informs therapeutic decisions. In contrast, the diagnosis and staging of invasive carcinoma involve a different set of investigations, including advanced imaging (e.g., MRI and CT) to determine the extent of local invasion and metastatic spread, which are not applicable to the pre-invasive stage discussed herein [33,34].

### 4.2. The Role of Colposcopy and Biopsy in CIN Diagnosis

The cornerstone of CIN diagnosis lies in the histopathological examination of a tissue sample obtained via biopsy. Colposcopy plays an indispensable role in this process by allowing for the visual identification of the most suspicious areas for targeted biopsy following an abnormal screening test [32]. This approach ensures that the definitive diagnosis is based on clear and objective histological evidence, which is crucial for developing an appropriate treatment plan and assessing the patient’s risk of progression [31]. It is important to note that CIN is typically asymptomatic, and the symptoms described in the literature, such as abnormal vaginal bleeding or discharge, are associated with invasive cancer, not with precancerous lesions themselves [33]. While most colposcopies are performed in an outpatient setting, complex cases may require examination under anesthesia by a specialized team [34].

### 4.3. Cervical Cancer Screening

The second report of the UK National Screening Committee [35] proposed a revised definition of screening. The original definition, adopted from Wald (2001), described screening as “the systematic application of a test or inquiry to identify individuals at high risk of developing a specific disorder in order to justify further investigation or direct preventive actions, among persons who have not sought medical care because of symptoms of that disorder” [36].

The revised definition emphasized that the screening should be conceptualized as risk reduction. Accordingly, screening programs were redefined as “risk-reduction programs”. For instance, screening for diabetic retinopathy would be reframed as a “vision preservation in diabetes, risk-reduction program”, while breast cancer screening would be considered a program for “reducing the risk of death from breast cancer”. These broader terms apply not only to the screening but also to clinical interventions and treatments [37]. The definition was further refined by the UK National Screening Committee in the Journal of Medical Screening [37], shifting the emphasis from mass testing to patient-centered benefits, by framing screening as “justifying further investigation or direct preventive treatment” [38].

In practice, the primary aim of screening is to detect disease at a stage when it is potentially curable. Screening tests are recommended by physicians after a thorough discussion with the patient, weighing both potential benefits and risks. Programs may target populations with a high prevalence of disease, while individual recommendations consider personalized risk factors [39]. Screening applies to asymptomatic individuals with the purpose of identifying conditions before clinical manifestations appear.

A screening test, sometimes referred to as medical surveillance, is a procedure performed in a defined, asymptomatic population (or subgroup) to evaluate the likelihood of disease. However, with few exceptions, screening does not establish a definitive diagnosis; abnormal results require confirmatory diagnostic testing [40].

Established screening tools include the Papanicolaou [41,42] and HPV testing [43,44,45,46]. The overarching goal is to reduce morbidity and mortality by enabling early detection when treatment is most effective.

### 4.4. Cervical Cancer Screening Program

According to data from the European Cancer Information System [47], in 2022, there were 30.447 new cases and 13.437 estimated deaths from cervical cancer in Europe. Of these, 11.1% of new cases and 13.43% of deaths were recorded in Romania. Despite this burden, HPV vaccination coverage in Romania remains low.

The national HPV vaccination program, initially introduced in 2008 and offered free of charge to girls aged 10–11, was met with significant resistance. Relaunched in 2009 alongside an informational campaign, uptake remained poor, leading to the suspension of the program for nearly a decade [48]. The program resumed in 2019 [49], targeting girls aged 11–14 years, later expanded to ages 11–18, with vaccination available through family physicians based on parental request.

In 2023, Order of the Ministry of Health No. 3.120 expanded eligibility further: free vaccination is now available for girls and boys aged ≥11 and <19 years, and women aged 19–45 years benefit from 50% cost reimbursement [50].

**Figure 5:**
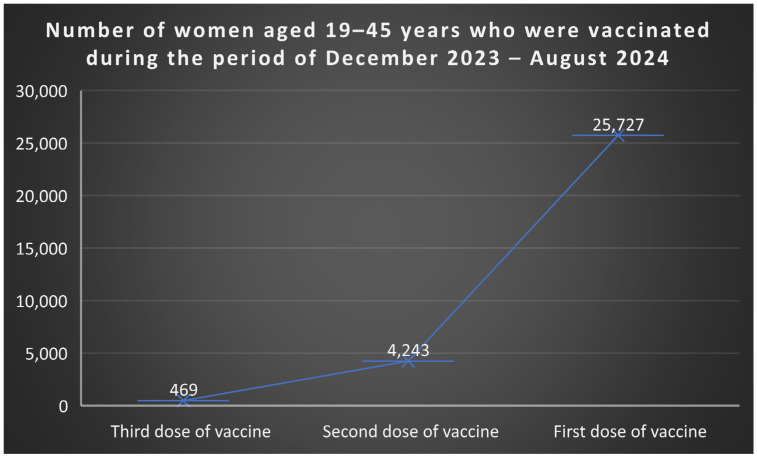
Number of women aged 19–45 years who were vaccinated during the period of December 2023–August 2024. Source: author, based on available data [50].

**Figure 6:**
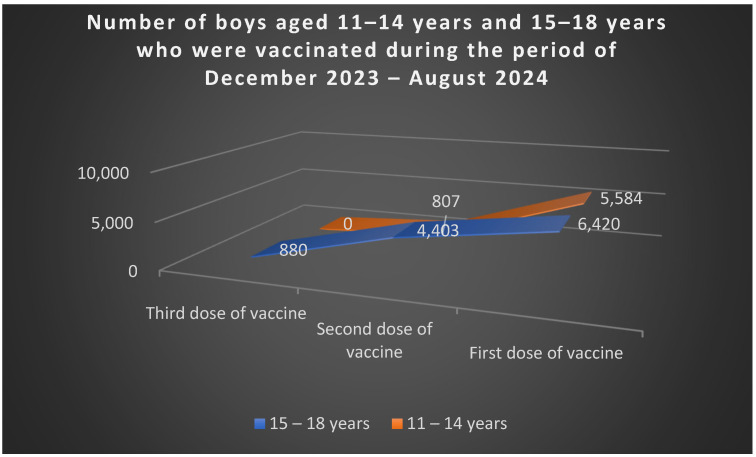
Number of boys aged 11–14 years and 15–18 years who were vaccinated during the period of December 2023–August 2024. Source: author, based on available data [50].

According to data from the National Institute of Public Health [51], between December 2023 and August 2024, among women aged 19–45 years, 25.727 received the first vaccine dose, 4.243 received the second, and 469 completed the third dose (Figure 5). Among boys, 5.584 aged 11–14 received the first dose, and 807 completed two doses; in the 15–18 age group, 6.420 received the first dose, 4.403 two doses, and 880 all three doses (Figure 6).

The European Cancer Organization’s 2024 report, Putting HPV on the Map: The State of HPV Prevention Programmes in the WHO European Region [51], highlights the critical role of HPV vaccination and screening in reducing cervical cancer incidence. While screening programs have reduced mortality in high-income countries, progress remains limited in resource-constrained settings, where adequate infrastructure and follow-up care are lacking [52].

Currently, many European countries are transitioning from cytology-based to HPV-based screening, following WHO recommendations [2]. In 2020, the WHO adopted the Global Strategy for the Elimination of Cervical Cancer [53], with three central targets:

1. Vaccination: Ninety percent of girls fully vaccinated against HPV by age 15.
2. Screening: Seventy percent of women screened with a high-performance test by age 35 and again by age 45.
3. Treatment: Ninety percent of women with precancerous lesions and ninety percent of those with invasive cervical cancer receiving appropriate treatment.

The European Beating Cancer Plan [54] reinforces these goals, aiming to vaccinate at least 90% of the target female population and significantly increase HPV vaccination coverage among boys by 2030. Achieving this requires sustained political commitment, funding, and healthcare system capacity, focusing on four priority areas: prevention, early detection, diagnosis and treatment, and quality of life [55].

According to the European Cancer Organization’s 2024 report, Romania’s incidence and mortality rates in 2020 were 22.6 and 9.6 per 100.000 women, respectively. The national immunization program, reintroduced in 2020, targeted girls aged 11–14, though vaccination coverage data were initially unavailable. The national cervical cancer screening program [56], ongoing since 2012, is cytology-based, targeting women aged 25–64 at 5-year intervals.

**Figure 7:**
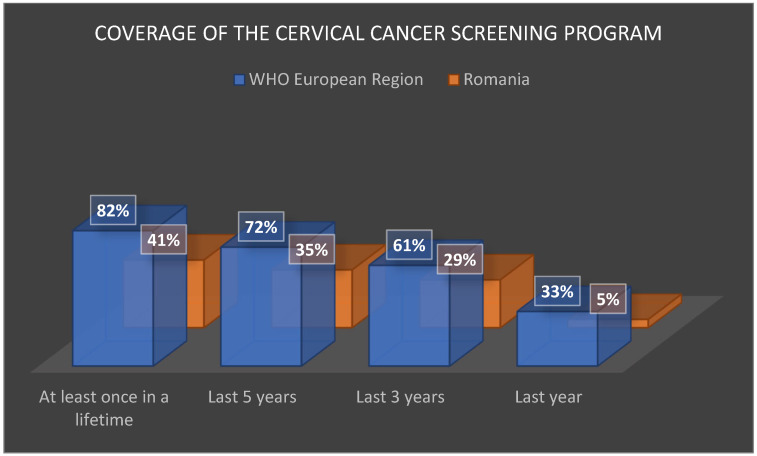
Coverage of the cervical cancer screening program. Source: author, based on available data [57].

However, its performance remains modest: coverage rates are approximately half the European average. INSP statistics [57] show coverage of only 5% in the past year (vs. 33% across Europe), 29% in the past 3 years (vs. 61%), 35% in the past 5 years (vs. 72%), and 41% at least once in a lifetime (vs. 82%) (Figure 7).

Participation could be improved through large-scale educational campaigns and better involvement of primary healthcare services. These efforts, combined with expanded vaccination programs, are essential steps toward reducing cervical cancer incidence and mortality in Romania.

### 4.5. Limitations and Future Directions

The limitations of the evidence base include heterogeneous study designs, variable follow-up durations, and lack of primary patient-level data. While these factors limit quantitative synthesis, the descriptive approach provides valuable insight into real-world clinical practice. Future research should focus on prospective studies, larger cohorts, integration of molecular and genomic markers for improved risk stratification, and evaluation of long-term follow-up strategies.

Several barriers affect the consistent application of evidence-based CIN management in Romania. Key obstacles include the following:

- Resource constraints: Limited access to high-performance HPV testing and colposcopy in some regions restricts guideline-concordant management.
- Training and expertise gaps: Variability in clinician experience may lead to more aggressive treatment than recommended.
- Patient compliance: Successful surveillance depends on reliable follow-up, yet low screening participation and limited engagement in HPV vaccination programs can reduce effectiveness.

Addressing these challenges is critical for improving adherence and ensuring optimal, guideline-based care.

## 5. Conclusions

This study emphasizes the strong concordance between local medical practice and international recommendations, while also underlining the critical importance of a personalized approach tailored to individual patient profiles. Optimal management of cervical dysplasia requires patient education, careful monitoring of lesions with regression potential, and the continuous professional development of medical personnel in line with current guidelines. Furthermore, the standardization of therapeutic decisions through unified protocols, alongside the digitization of patient follow-up, can significantly improve both the efficiency and safety of cervical dysplasia management.

Accordingly,

1. The management of cervical dysplasia must be personalized, considering lesion severity, reproductive desires, and the patient’s capacity to adhere to medical recommendations.
2. Compliance with ASCCP and ESGO guidelines ensures standardized management, with measurable benefits for prognosis.
3. Rigorous monitoring and judicious decisions regarding excisional or ablative treatment are crucial to prevent progression to invasive cervical cancer.

A streamlined diagnostic pathway for cervical precancerous lesions remains indispensable for accurate grading and guiding therapeutic strategies. Screening methods such as the Papanicolaou test and HPV testing are fundamental to early detection, while colposcopy and biopsy provide the essential histopathological confirmation required for treatment planning [58]. It is critical to reiterate that advanced imaging plays no role in the management of CIN and is reserved exclusively for the staging of established invasive carcinoma. The Pap smear continues to be a cornerstone in the prevention and early diagnosis of cervical neoplasia. When integrated with HPV testing and subsequent investigations, it constitutes a vital tool in safeguarding women’s reproductive health [59].

Over recent decades, evidence from the literature has consistently demonstrated that the early diagnosis and treatment of cervical dysplasia represent one of the most effective advances in reducing cervical cancer-related morbidity and mortality. HPV testing further reveals a roughly equal distribution of high-risk and low-risk strains, while genotyping allows the identification of women at greatest risk. Thus, HPV strain detection serves as an important tool for patient risk stratification. In turn, coloscopic examination facilitates the localization, characterization, and grading of suspicious lesions identified through cervical cytology, guiding targeted biopsies and consolidating its role as a key element in the cytology–colposcopy–histological triad.

Nonetheless, this study is limited by its nature and sample size, which may constrain generalizability. Future research should include larger, prospective cohorts and explore the integration of HPV genotyping, digital follow-up tools, and expanded vaccination strategies to further optimize prevention and management.

## Figures and Tables

**Table 1:** Comparison of study findings with ASCCP 2019 and ESGO 2020 guideline recommendations.

CIN Grade	Our Findings	ASCCP 2019	ESGO 2020
CIN 1	High regression, low progression	Observation	Conservative
CIN 2	Mixed behavior	Individualized	Selected conservative
CIN 3	No regression	Treatment	Treatment

**Table 2:** Distribution of dysplasia grades and therapeutic approaches reported in the analyzed articles.

Grade CIN	Number of Articles	Active Monitoring	Excision/LEEP	Adjuvant Vaccination	HPV Monitoring/Cytology	Historical Modeling/Risk Analysis
CIN 1	2	✓		✓	✓	
CIN 2	5	✓	✓		✓	
CIN 3	2		✓			
CIN 2/3	5	✓	✓		✓	✓
CIN 1–3	4		✓			✓
CIN 2+	2		✓		✓	

**Table 3:** Type of progression and estimated rate for CIN1, CIN2, CIN2/3, and CIN3.

Grade CIN	Type of Evolution	Estimated Rate or Conclusion
CIN 1	Surveillance vs. conization	Low recurrence; possible upgrading and clearing; ~70% regression within 1–2 years
CIN 2	Active surveillance	Progression to CIN 3 in approximately 8–16% (variable), influenced by HPV status and vaccination
CIN 2/3	Excisional (LEEP and conization)	Recurrence rate of ~5–7% at 5 years; persistently increased risk in HPV-positive cohort individuals
CIN 3	Post-treatment (follow-up long)	Elevated risk of anogenital cancer (anal, vaginal, and vulvar) persists for years; decreases over time but remains significant

**Table 4:** Presence of the lesions studied in the 20 analyzed articles.

Nr.	Author and Year	Lesions Studied	Treatment	Other Reported Interventions	Regression Rate	Progression/Recurrence	Other Observations
1.	[11]	CIN 1–3	LEEP, conization observation	Decision algorithm	Var. (CIN1 > 50%)	Depends on grade	General management approach for CIN
2	[12]	CIN 1	Not reported	Adjuvant HPV vaccination	~80%	~10–15%	Vaccination reduces progression
3	[13]	CIN 2	Observation	No initial treatment	16.7%	3.6%	Short-term safe observation
4	[14]	CIN 2/3	LEEP	HPV, surgical margins	N/A	~6–9%	Positive margins + HPV = risk ↑
5	[15]	CIN 2/3	Excision	HPV test, colposcopy	N/A	6.8% (↑ la HPV 16/18)	HPV 16/18 = HR ~5
6	[16]	CIN 1–3	Not reported	Analysis of risk factors	N/A	N/A	Etiologic and genetic context
7	[17]	CIN 2	Observation	HPV vaccination	Unspecified	↓ Within the vaccinated group	The vaccine protects against progression
8	[18]	CIN 2/3	Excision	Postoperative HPV	N/A	~8–12%	Persistent HPV = risk ↑
9	[19]	CIN3	Conization	HPV	84% clearance	<0.2%	Effective post-operative screening
10	[20]	CIN 1	Conization	Histology post-op	N/A	Significant upgrading	Some CIN1 = CIN2 underdiagnosed
11	[21]	CIN 2/3 (<25 years)	Conization	HPV, margins	71.8%	8% CIN2/6.4% CIN3	Validation of surveillance in young women
12	[22]	CIN 2+	Not reported	p16/Ki-67	N/A	Predictive risk CIN3+ ↑	Dual stain = efficient triage
13	[23]	CIN 2/3	Excision	HPV post-op	N/A	~0.65% vagin. cancer	Risk ↑ >50 years, HPV 16
14	[24]	CIN 1–3	Not reported	Statistic model	N/A	Progressive estimates	Predictive model screening
15	[25]	CIN 3	Conization	HPV, margins	N/A	~4.2% recurrence	Risk ↑ positive margins
16	[26]	CIN 2	Observation	Genotype HPV	N/A	↑ la HPV 16/18	HPV = predictor of progression
17	[27]	CIN 2	Observation vs. excision	Comparative	N/A	↓ Post-excision recurrence	Benefit of excisional treatment
18	[28]	CIN 2	Observation	No treatment	~40%	~5–7%	Recommendation for active monitoring
19	[29]	CIN 2+	Observation	Persistent HPV	N/A	Confusion progression vs. coexistence	CIN3 can coexist with LSIL
20	[30]	CIN 1–3	Management algorithm	ASCCP guidelines	N/A	Stratified risk	Update practice guideline

CIN—cervical intraepithelial neoplasia; LEEP—loop electrosurgical excision procedure; HPV—human papillomavirus; ASCCP—American Society for Colposcopy and Cervical Pathology; N/A—not applicable.

**Table 5:** Clinical outcomes of CIN grades 1–3: regression, progression, and recurrence across reviewed studies.

CIN Grade	Studies (Author, Year)	Regression Rate	Progression/Recurrence	Key Points/Notes
CIN 1	[11,12,16,20,24,30]	~80% [12]; Var. (CIN1 > 50%) [11]; N/A [16,24,30]; N/A [20]	~10–15% [12]; significant upgrading [20]; depends on grade [11]; N/A [16,24,30]	High spontaneous regression; active surveillance recommended; some CIN1 may be underdiagnosed as CIN2
CIN 2	[13,17,26,27,28]	16.7% [13]; Unspecified [17]; N/A [26]; ~40% [28]; N/A [27]	3.6% [13]; ↓ post-excision recurrence [27]; 5–7% [28]; ↑ la HPV 16/18 [26]	Heterogeneous outcomes; influenced by HPV genotype, patient age, and vaccination; active surveillance possible in selected patients
CIN 2/3	[14,15,18,21,23]	N/A [14,15,18,23]; 71.8% [21]	~6–9% [14]; 6.8% [15]; ~8–12% [18]; 8% CIN2/6.4% CIN3 [21]; ~0.65% vaginal cancer [23]	Mostly treated with excisional procedures; recurrence associated with HPV persistence and positive margins
CIN 3	[19,25]	84% clearance [19]; N/A [25]	<0.2% [19]; ~4.2% recurrence [25]	No spontaneous regression; excisional treatment required; long-term cancer risk persists; risk increased with positive margins and HPV

## Data Availability

The original contributions presented in this study are included in this article. Further inquiries can be directed to the corresponding authors.
